# Need for shared internal mound conditions by fungus-growing *Macrotermes* does not predict their species distributions, in current or future climates

**DOI:** 10.1098/rstb.2022.0152

**Published:** 2023-08-28

**Authors:** Colleen L. Seymour, Judith Korb, Grant S. Joseph, Richard Hassall, Bernard W. T. Coetzee

**Affiliations:** ^1^ South African National Biodiversity Institute, Kirstenbosch Research Centre, Private Bag X7, Claremont 7735, South Africa; ^2^ FitzPatrick Institute of African Ornithology, Department of Biological Sciences, University of Cape Town, Rondebosch 7701, South Africa; ^3^ Faculty of Biology, Evolutionary Biology and Ecology, University of Freiburg, D-79104 Freiburg, Germany; ^4^ UK Centre for Ecology and Hydrology, Maclean Building, Benson Lane, Crowmarsh Gifford, Wallingford, Oxfordshire OX10 8BB, UK; ^5^ Department of Zoology and Entomology, University of Pretoria, Private Bag 20, Hatfield, 002, South Africa

**Keywords:** African termites, climate change projections, *Termitomyces* fungus-culturing, fungus farming, species distribution models, obligate symbiotic relationship limits on species

## Abstract

The large, iconic nests constructed by social species are engineered to create internal conditions buffered from external climatic extremes, to allow reproduction and/or food production. Nest-inhabiting eusocial Macrotermitinae (Blattodea: Isoptera) are outstanding palaeo-tropical ecosystem engineers that evolved fungus-growing to break down plant matter *ca* 62 Mya; the termites feed on the fungus and plant matter. Fungus-growing ensures a constant food supply, but the fungi need temperature-buffered, high humidity conditions, created in architecturally complex, often tall, nest-structures (mounds). Given the need for constant and similar internal nest conditions by fungi farmed by different *Macrotermes* species, we assessed whether current distributions of six African *Macrotermes* correlate with similar variables, and whether this would reflect in expected species' distribution shifts with climate change. The primary variables explaining species’ distributions were not the same for the different species. Distributionally, three of the six species are predicted to see declines in highly suitable climate. For two species, range increases should be small (less than 9%), and for a single species, *M. vitrialatus*, *‘*very suitable’ climate could increase by 64%. Mismatches in vegetation requirements and anthropogenic habitat transformation may preclude range expansion, however, presaging disruption to ecosystem patterns and processes that will cascade through systems at both landscape and continental scales.

This article is part of the theme issue ‘The evolutionary ecology of nests: a cross-taxon approach’.

## 1. Introduction

**Figure 1.**
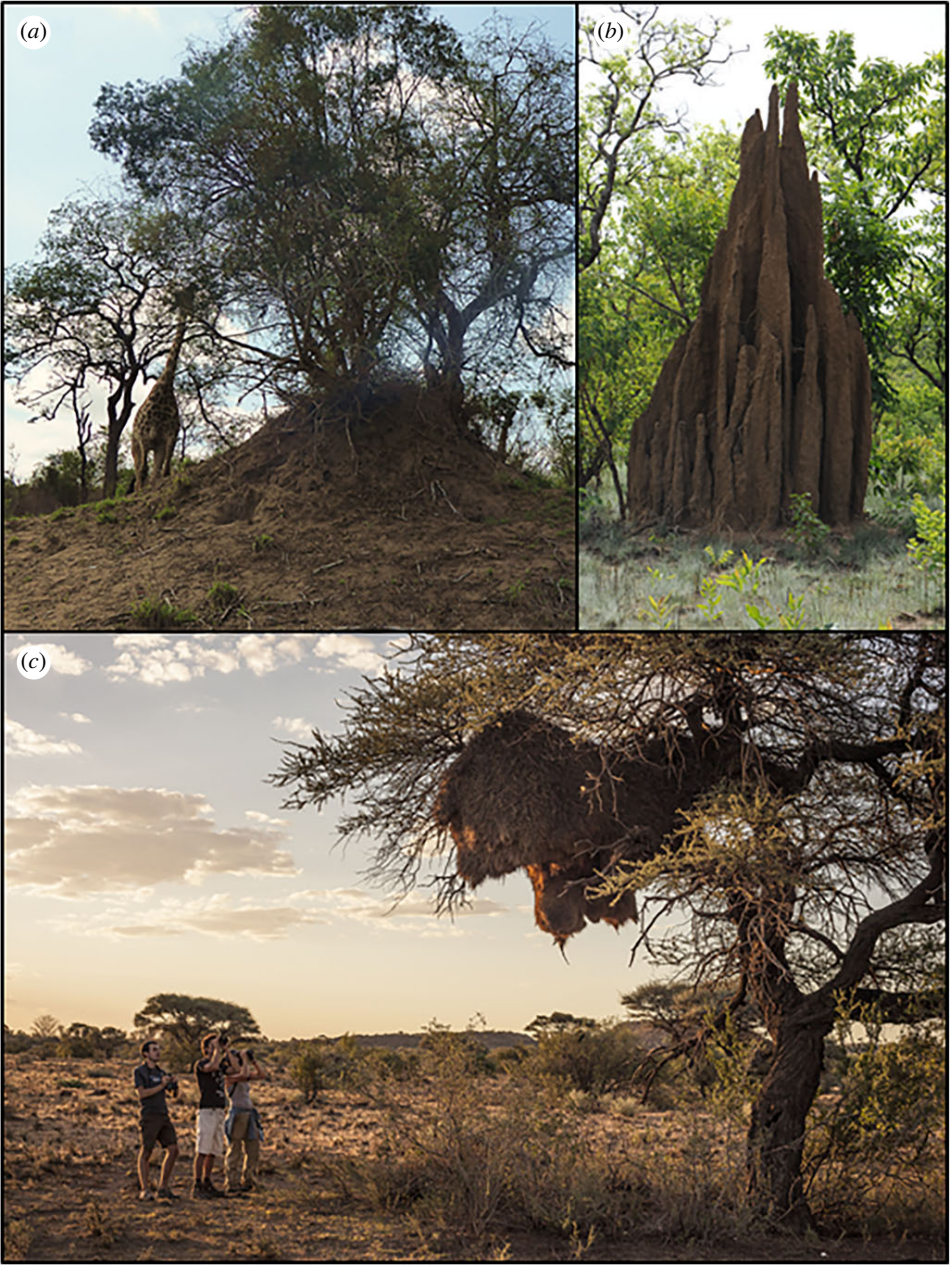
Nests, for example mounds of (*a*) *Macrotermes falciger* (note size, with adult giraffe in background); (*b*) *Macrotermes bellicosus* (which can reach 7 m high [1]) and (*c*) nests of sociable weavers (*Philetairus socius*) are often iconic structures, built to create suitable ambient conditions for breeding and/or feeding. Photo credits (*a*) Colleen Seymour; (*b*) Judith Korb; (*c*) Alexander Vaz.

The large nests constructed by social species not only produce remarkable structures across landscapes (figure 1) but also influence biodiversity [2–4]. These nests are often intricately engineered to create internal ambient conditions quite different from the climatic extremes of their surrounding environment, to enable breeding (e.g. among ants, termites, bees and some bird species) and food production (e.g. by ants, beetles and termites). At least 40 million years before humans took up agriculture, three insect groups (termites, ants and beetles) evolved the ability to grow fungi as food [5,6]. These groups are unable to fully break down plant matter, so they employ fungi to do so for them by providing the fungus with plant matter; the fungus is then either consumed or fed to their broods [7,8]. Fungus cultivation evolved *ca* 62 Mya [9] among forest-dwelling termites [10] and allowed termites to exploit an array of plant substrates through decomposition of dead plant material [11]. Mound-building termites are industrious architects and builders, working ceaselessly to maintain optimal conditions within their nests (hereon referred to as mounds), because fungus growing requires a narrow range of climatic conditions [12,13], like that of the forests where fungus growing by termites evolved [10]. The mounds of *Macrotermes* (Termitidae: Macrotermitinae), a genus occurring through Africa and south-eastern Asia, can be particularly large and impressive, with some reaching metres high and tens of metres wide [14–16]. The mounds can be ancient. Queens of some *Macrotermes* species have been found to live a couple of decades [17,18], suggesting the colonies last for at least that length of time. The mounds themselves are abandoned and recolonized repeatedly over time, and have been found to be centuries old, with some over 2000 years old [19].

The complex mound architecture and social behaviours allow termites to fine-tune internal nest conditions [16,20] for their *Termitomyces* fungal symbionts, buffering against environmental variability and predation [21]. The internal nest environment needs to be kept within a narrow temperature range of a few degrees, over days or years [20,22–25], with humidity near saturation, yet with sufficient gaseous exchange to avoid a build-up of CO_2_ [16,26,27]. Fungus-growing termite species are monophyletic, implying that fungus cultivation evolved only once. Reversal to non-fungus cultivation apparently has not occurred [28], highlighting the evolutionary and ecological success of this strategy.

Erpenbach & Wittig [29] and Bignell & Eggleton [30] give useful overviews of the key role of termites in African ecosystems. They are often key drivers of landscape heterogeneity and diversity through impacts on pattern and process [31]. The fungus-growing genus *Macrotermes* are prominent ecosystem engineers in African systems that can represent a large proportion of the total soil macrofauna, and in savannahs, their biomass compares to that of ungulates [32]. Through their mound-building activities, *Macrotermes* change the distribution of water [33] and nutrients [34] in soils. Active mounds can be regularly spaced [35–39], creating habitat heterogeneity that contributes to plant [40–42], invertebrate [43], bird [3], mammal [4] and functional group diversity [44,45], patterns of grazing and browsing [14,46,47], productivity [48], drought amelioration [49,50], and the interplay between top–down and bottom–up processes in ecosystems [51]. Were termites to disappear, consequences for other biota may only unfold slowly, but would ultimately cascade through the ecosystem.

The obligate association with fungi that have narrow environmental requirements might be a mixed blessing for fungus-growing termites [52]. Although the fungus has extended the niche of Macrotermitinae, making them the dominant termite species, particularly in drier regions [33,52], the fungus' strict requirements may also constrain termite species’ distributions [24,26,52,53]. Termites can change the architecture of their mounds in response to external conditions. For example, *Macrotermes bellicosus* in northern Ivory Coast increased the surface area to volume ratio of their mounds in response to removal of the surrounding trees in forest habitats [24]. They may also thicken walls [53], or open holes in the mound surface [54].

A single established termite nest contains only a single *Termitomyces* symbiont strain (which can be considered analogous to a crop cultivar selected by humans) at any one time, but some termite species have multiple strains within a region, which might offer flexibility for conditions needed in the nest interior [25,28,55]. For example, *Macrotermes subhyalinus* and *M. michaelseni* in Tsavo, Kenya, can cultivate more than one *Termitomyces* fungal symbiont [56,57]. The different symbionts are associated with different temperature conditions, with one *Termitomyces* consistently found in ‘hotter’ mounds, and mound architecture associated with the particular *Termitomyces* present [56]. The variation allowed by cultivation of different strains of *Termitomyces* is limited to only a few degrees, however. Other species, like *M. bellicosus* in Ivory Coast, seem to have only a single strain of fungal symbiont [25,28]. This might help explain why its distribution is limited largely to the savannah, with lower mound densities in forest habitats [52].

Given the temperature sensitivity of the obligate fungal symbiont, climatic conditions are likely to be key to fungus-growing termite species' distributions. The narrow requirements of *Termitomyces* could mean that the distributions of all *Macrotermes* are determined by similar climatic variables. Alternatively, divergent characteristics unique to a particular species, like mound structure and particular species of *Termitomyces*, may allow adaptation to climate, and hence distribution. Habitat structure is important: changes in vegetation cover may leave mounds exposed to extreme temperature fluctuations too costly for termites to modulate [58,59]. For *Macrotermes* mounds in northern Kruger National Park, South Africa, there were few active mounds below a certain level of vegetation cover, and temperatures on mound surfaces were extreme [60], suggesting additional thermal challenges for the colony posed by lack of shade. In more arid or open systems, water and humidity could limit species’ occurrence, as fungus cultivation requires high humidity. In forests, the relatively high temperatures (*ca* 30°C) required within the nest may also limit species' presence. For example, in Ivory Coast forests, which are relatively cooler than savannahs, the trade-off between respiratory gas exchange and thermo-insulation seems to restrict *M. bellicosus* to the savannah and open forest stands or forest edges [26,61]. In savannah, *M. bellicosus* nest temperatures are maintained at *ca* 30°C, with daily and annual fluctuations < 2°C [20,23,26]. However, in open forest stands, even in large colonies, temperatures drop to 26–28°C, and although daily and annual fluctuation is small [24,26], these temperatures are below the optimum for growth of both fungal symbiont and termites [20,62], with negative impacts on reproductive success of forest colonies [61]. The most challenging period is during colony establishment, when the colony is still small. Small (incipient) colonies are less buffered against adverse ambient conditions because they have fewer individuals and thus lower ability to maintain the metabolism needed for constant conditions [23]. In addition, the mound structure is still fragile with less buffering ability against the environment, so temperature variability within *Macrotermes* mounds decreases with increasing colony size [23].

The extreme sensitivity to variation and narrow range of tolerable temperatures for *Termitomyces* may constrain the various species of *Macrotermes* similarly, such that the different species’ distributions are determined largely by the same climatic variables. Alternatively, a combination of strategies that allow termites to alter mound architecture, differences in nest microclimates provided by vegetation, variation in colony sizes, climatic conditions while nests are in their incipient stages (and therefore most vulnerable), and the small, but perhaps crucial differences in fungus strains' temperature tolerances may mean that these termite distributions are determined by different climatic variables, now and in future.

Here, we focus on African *Macrotermes* as there is insufficient distributional information for Asian genera, and there are many cases of unresolved species status within other genera in the Macrotermitinae (e.g. *Odontotermes* and *Microtermes;* [63]), where species identification is difficult and often wrong. We aimed to identify environmental factors associated with the distribution of fungus-growing termites using available geographical distribution information and species distribution models (SDMs). Although museum data can provide much information on species presence, limitations include inaccuracies in locations and nomenclature, relatively few records for some taxa, and spatial, temporal, taxonomic and environmental collecting biases [64]. By characterizing species' ecological niches, SDMs can extrapolate from incomplete datasets to estimate occurrence in unsampled areas. Globally, marked climate-associated changes in faunal and floral distribution are underway [65]. Although the genus *Macrotermes* arose *ca* 47 Mya (22–84 Mya credibility intervals; [9]), when the climate was warmer than today, the Earth has slowly cooled in the intervening period, and current models of anthropogenic climate change predict rapid heating, to which modern termites and their symbionts may not be adapted. To better understand current and future distribution of *Macrotermes*, we apply a bioclimatic envelope modelling approach to assess (i) whether, because *Macrotermes* are limited by their symbiotic fungi that have such similar needs, their distributions are determined by the same climatic variables, and (ii) whether expected changes in species distributions with climate change will be similar.

## 2. Methods

### (a) Species data

**Figure 2.**
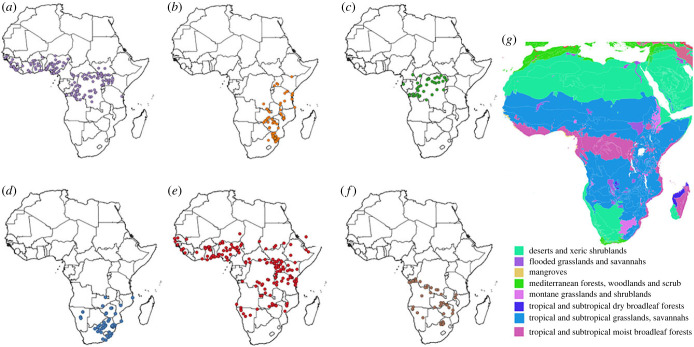
Point data used to model species distributions for (*a*) *Macrotermes bellicosus,* (*b*) *M. falciger*, (*c*) *M. muelleri,* (*d*) *M. natalensis,* (*e*) *M. subhyalinus* and (*f*) *M. vitrialatus*; along with the (*g*) biomes map for Africa based on Olson *et al*. [67], allowing assessment of the vegetation types in which each species occurs.

Owing to confusion regarding the status of some species of *Macrotermes*, we based our species distribution data on records for *Macrotermes* covered in Ruelle [66]. There were insufficient species occurrence records to allow production of reasonable SDMs for six of the 12 species included in Ruelle [66]. Therefore, we limited our analyses to the remaining six species: *M. bellicosus, M. falciger, M. muelleri, M. natalensis, M. subhyalinus* and *M. vitrialatus* (figure 2; species information, electronic supplementary material, S1). Ruelle [66] represents the most comprehensive compilation of sampled specimens to date across multiple museum and private collections. We constructed a database (electronic supplementary material, S2) using Google maps™ to extract latitude and longitude from reported locations (often described only as distances from towns along certain roads). These data were supplemented with additional records from research papers for which there was either an indication that an expert in *Macrotermes* taxonomy was an author or had been consulted. These data are therefore slightly older than the climatic conditions used in our SDMs. Nevertheless, the models gave sensible approximations of termite current distributions and estimates of future suitable climate envelopes. Species data were thinned to presence at sites using a resolution of 2.5 arcminutes (approx. 5 km × 5 km). We used this coarser resolution because the age of *Macrotermes* occurrence records increases geographic uncertainty and therefore we opted not to run the models at the finest spatial scale.

### (b) Climatic variables

We identified climatic variables most likely to influence species distributions: mean annual temperature, minimum temperature, maximum temperature, diurnal temperature range, and difference between annual maximum and minimum temperatures (to try to capture the range of temperatures to which species are exposed), mean annual precipitation (which owing to the climate of much of the area, we assume to equate to rainfall) and precipitation seasonality (see electronic supplementary material, S3). We obtained data for these from WorldClim 1970–2000 at a resolution of 2.5 arcminutes [68]. We also included biome (descriptions given electronic supplementary material, S4) in our analyses using the Terrestrial Ecoregions of the World dataset [67].

### (c) Species distribution models

Environmental suitability across Africa was established using a boosted regression tree framework [69]. Given that we used presence-only data, an absence of a species from a particular locality does not confirm that it is absent from that point, only that it has not been recorded there. To address this issue, we used a pseudo-absence based approach by which absences were selected randomly from across the continent of Africa. Selection was weighted using the density of Insecta records from the Global Biodiversity Information Facility in an effort to select absences from regions where insects were more likely to be recorded ([70]; electronic supplementary material, figure S1). We selected the same number of absences as presences following recommendations from Barbet-Massin *et al*. [71]. To avoid issues related to collinearity, we calculated the Pearson's correlation coefficient between all pairs of variables. Brun *et al*. [72] suggest that a correlation coefficient of 0.7 restricts collinearity-driven effects and none of the correlation coefficients calculated between variables significantly exceeded this threshold, with a maximum correlation coefficient of 0.72 (electronic supplementary material, S5).

We selected 100 sets of pseudo-absences using this approach and ran 100 models for each species. Suitability and the relative influence of each environmental variable were established by calculating the mean of suitability predictions at each site and calculating the mean percentage relative influence for each variable using results from all models, similar to methods used in Purse *et al*. [73]. Model predictive performance was established using the area under the receiver operating characteristic curve (AUC), which evaluates the ability of models to discriminate between sites where species are present and absent. A value of 1 indicates perfect discrimination and a value of 0.5 indicates that discrimination is no better than random. The AUC was obtained using the cross-validation approach outlined in Elith *et al*. [68] and the mean and standard deviation of AUC values from 100 models were used to evaluate model performance.

To select learning rates and tree complexity, we initially ran models varying tree complexity from 1 to 7 and learning rate from 0.001 to 0.003. The tree complexity with the lowest predictive deviance and learning rate resulting in models fitted using more than 1000 trees was selected for each species [69].

## 3. Species distribution with climate change

Suitability in 2050 was predicted using all model predictions for each species based on climatic conditions under two different climate scenarios that represent medium and medium to high range of plausible future scenarios (SSP245, SSP370; [74]). The more extreme scenarios of SSP126 and SSP585 were not considered in this analysis. These data for climatic variables predicted using the CNRM-CM6-1 climate model were obtained from WorldClim (resolution 2.5 arcminutes; [68]). To identify changes in ‘very suitable’ climate conditions, we quantified the change in areas up to 15% below the current maximum suitability values for each species and limited predictions based on distribution data used in the models (figure 2).

We then excluded biologically irrelevant areas, i.e. areas from which species are likely to be excluded because of the need for horizontal and vertical linkages with other species and resources in ecosystems, or phylogenetic history [75]. We did this for both current distributions and future projections, where there is no feasible likelihood of current occupancy or future colonization (see electronic supplementary material, figures S2,i–vi, for area comparisons across all six species).

All analysis was carried out in R CRAN [76]. Models and predictions were generated using the *gbm.step* and *predict* functions in the dismo R package [77].

## 4. Results

### (a) Environmental variables explaining species distributions

The models for all species except *M. natalensis* can be considered ‘very good’ (AUC > 0.8) or better (table 1). The model for *M. natalensis* can be considered ‘fair’ (AUC 0.6−0.7). For some species, a single variable explained most of the suitability: minimum temperature was a major explanatory variable for *M. bellicosus*, *M. muelleri* and *M. subhyalinus* and biome for *M. vitrialatus.* The remaining two species were mostly associated with precipitation seasonality and/or amount, with biome also important for *M. falciger* (table 1).

**Table 1.** Mean relative influence (%) of climatic variables and biome on suitability for different African *Macrotermes* for which we had sufficient data. Cells are coloured darker to indicate higher influence of those variables for a particular species. Values in bold reflect the most important variables for the species under consideration. Variables in italics describe the model.

variable	*M. bellicosus*	*M. falciger*	*M. muelleri*	*M. natalensis*	*M. subhyalinus*	*M. vitrialatus*
number of data records used	147	79	42	47	174	45
current predominant vegetation types	grass, shrub, tree savannah, woodland	woodland	forest	savannah/ woodland	grass, shrub, tree savannah, woodland	no forest, open woodland and savannah
mean annual temperature	1.76	10.40	4.54	13.90	4.73	10.70
min temperature	**91**.**30**	13.5	**67**.**30**	6.68	**65**.**60**	5.18
max temperature	0.76	6.13	0.23	6.97	1.93	7.19
difference between min and max temperature	1.22	6.66	**25**.**30**	6.59	2.72	5.18
diurnal temperature range	1.39	5.54	0.84	12.10	3.12	7.85
annual precipitation	1.42	12.10	0.40	**22**.**40**	5.35	9.61
precipitation seasonality	1.71	**22**.**70**	0.86	**27**.**40**	13.10	**13**.**70**
biome	0.46	**22**.**90**	0.49	3.94	3.46	**39**.**50**
*number of trees (mean)*	2300	1625	1319	3766	2381	1800
*deviance explained*	0.94	0.66	0.94	0.26	0.86	0.74
*mean AUC*	0.99	0.87	0.99	0.68	0.97	0.89
*AUC s.d.*	0	0.03	0	0.06	0.01	0.03

### (b) Changes in distribution

**Figure 3.**
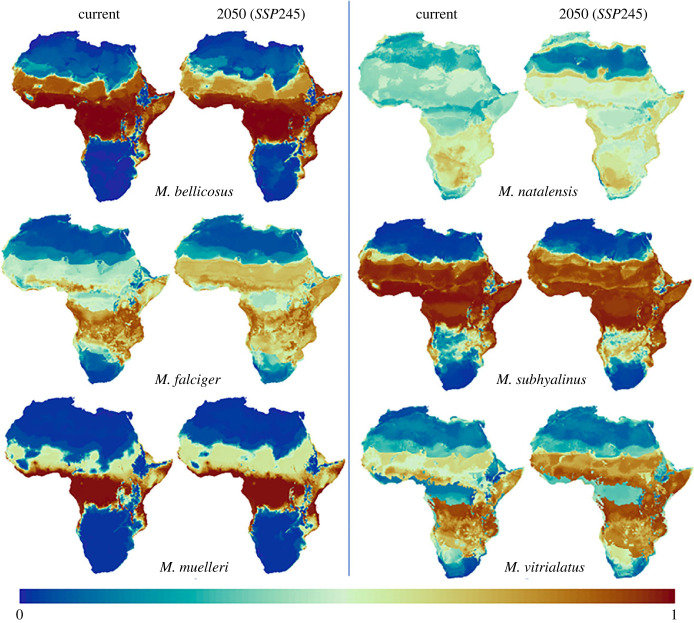
Predicted changes in climate suitability across Africa under medium socio-economic and emissions scenario (SSP245). Suitability ranges from 1 to 0, with 1 indicating highest possible suitability and 0 indicating lowest suitability (see colour key). Results for SSP370 did not differ significantly from SSP245 and can be found in the electronic supplementary material, figure S2.

All species are predicted to experience some change to their potential ranges under future climate scenarios (figure 3). Under the two medium and medium to high range of plausible future scenarios (SSP245, SSP370), *M. bellicosus* could lose much of its northern ‘very suitable’ climate, with a projected loss between 2 918 258 km^2^ and 2 935 827 km^2^ (29.5–29.6%). *M. falciger* is predicted to lose between 1 000 007 and 1 086 892 km^2^ (51%−55.5%) and *M. natalensis* between 133 233 and 168 303 km^2^ (17%−21%) of their ‘very suitable’ climate relative to current distributions. By contrast, impacts should be minimal for the forest species *M. muelleri*, which may even gain from 374 147 to 365 292 km^2^ (9.5−9.7%) of very suitable climate. *M. subhyalinus* is predicted to make a gain of 214 618–307 567 km^2^ (1.5−2.1%). *M. vitrialatus* could experience large expansion in ‘very suitable’ climate of between 1 802 405 and 1 819 174 km^2^ (63.7−64.37%). Shifts are depicted graphically in figure 3 (darker brown shading represents ‘very suitable’ climate). The extent of change needs to be interpreted with caution, however, and is discussed in more detail within §5, because the models are limited by geographical feasibility (e.g. expansion cannot be reasonably anticipated into disjunct areas separated by impassable geographical or habitat barriers).

## 5. Discussion

All *Macrotermes* species are constrained by the needs of their *Termitomyces* symbionts for internal nest conditions that maintain narrow temperature ranges, sufficient gaseous exchange to ensure low CO_2_ and optimum humidity (see review in [16]). Despite this, we find that distributions of different *Macrotermes* species are not driven by the same climatic variables, suggesting that other factors, like mound structure, differences in *Termitomyces* strains and habitat structure are also key. Consequently, the overall response of the genus to climate change is nuanced. There were greater changes to future range predicted in species with distributions south of the Tropic of Capricorn, where climatic impacts to vegetation, rainfall and temperature are predicted to be greatest [78]. Models predicted a decrease in area of highest suitability relative to current distributions for half of the species, with small gains for *M. muelleri* and *M. subhyalinus*. *M. vitrialatu*s, which the models predict could expand its range by greater than 60%, may be unlikely to do so, given that this species is restricted to a particular biome (woodland open woodland savannah) and so may not realize much range expansion owing to barriers presented by anthropogenic habitat transformation.

## 6. Current and future distributions

The most important factors currently limiting species' distributions included minimum temperature, seasonality of precipitation and biome. Despite precipitation being linked to productivity, it emerged as the key driver for only one species, *M. natalensis*, with combined seasonality and annual precipitation explaining almost 50% of its distribution, whereas for *M. bellicosus* and *M. muelleri* this value approached zero. Importantly, half the species modelled are currently constrained by minimum temperature. Yet this variable, despite being the key determinant of distribution today, is unlikely to be a constraint in the future, as no regions modelled here are predicted to experience a decrease in minimum temperature.

Given the complex range of responses suggested by our climate change models, we interpret the future prospects of *Macrotermes* for each species considered, focusing not only on distribution changes and identified drivers, but also on mitigating and exacerbating factors that influence both current and potential future ranges.

*M. bellicosus* is predicted to be mainly constrained by minimum temperature. This concurs with previous findings about its distribution patterns in Uganda [79,80] and Ivory Coast, where *M. bellicosus* is excluded from cooler forests [24,53]. Its current core habitat spans deciduous savannah, woodland and forest edges. By 2050, much of its habitable northern range will have shrunk (figure 3).

*M. falciger* presently ranges from eastern Africa, to south of the Tropic of Capricorn. Here again, models predict marked loss of the most suitable range, to less than a half. The apparent expansion of intermediately suitable distribution ranges across the Sahel is misleading: this region is disjunct from the current range and separated from current population by highly unsuitable areas, making the possibility of future colonization of this region unlikely. Given that seasonality of precipitation and biome explain nearly half of its current distribution, the loss of most suitable distribution across its current range is easily explained. Vegetation and precipitation, particularly in the south-central aspects of its range (southern Zambia, Zimbabwe and Mozambique), are predicted to change, with arid-adapted vegetation following a drying, warming trend in Köppen–Geiger climate-vegetation models ([78]; see electronic supplementary material, figure S3).

Our models predict that forest-adapted *M. muelleri*, limited by minimum temperature, and distributed in forests of the Congo basin, will be minimally impacted, possibly because temperature changes in this equatorial region will be less marked [78]. Nevertheless, habitat transformation of forest, woodland and savannah in Africa could threaten persistence of this species.

The models for *M. natalensis* fit the least well, and this may be because the data used to model their distributions may represent two distinct species. Phylogenetic analyses of samples from Kruger National Park (northeast South Africa) and Malawi suggest this may be the case [9]. Nevertheless the model projections are consistent with expectations for climate. *M. natalensis* favours open savannah and is the only species with a range predominantly outside the tropics, ranging from Namibia to the eastern aspects of South Africa. Its distribution is associated with precipitation, and to a lesser extent, temperature, and across much of its westernmost range, temperature and precipitation are predicted to undergo the greatest change for all *Macrotermes* modelled, leading to increasingly hot and arid systems [78]. Overall, ‘very suitable’ climate for *M. natalensis* decreases by *ca* 20%. The projection of increased potential climatic envelope in North Africa is unlikely to be ecologically relevant, as this area is separated from current *M. natalensis* range by unsuitable habitat.

Models suggests that the distribution of *M. subhyalinus* is mainly driven by minimum temperature (consistent with [79,80]). To a lesser degree, seasonality of precipitation also affects its distribution, which spans inter-tropical, equatorial savannahs. Predicted change in distribution is minimal, with a small contraction in suitability to the northern aspects of range offset by a slight increase in the south.

The species distribution of *M. vitrialatus* is mostly determined by biome*.* Its current distribution is south of the Congo rainforest, mainly in deciduous woodland and more open savannah. A geographical expansion of up to *ca* 60% is possible across eastern and northeastern Africa, but much of this is almost 2000 km away from its current range. Given that colonization depends on how biomes will change and whether habitat exists to allow colonization, it is difficult to assess what actual increases in distribution might be. Köppen–Geiger climate-vegetation models [78] predict a decrease in aridity in these areas. If deciduous woodlands and savannahs expand then expansion may be possible.

## 7. Potential mechanisms for mitigating against climate

Minimum temperature was identified as the major determinant of current range size for *M. bellicosus,* but observations over recent decades suggest that precipitation is also key (J. Korb 2022, unpubl. data). The loss of range size with climate change predicted by our models seems consistent with climate predictions of decreased precipitation and later onset of the rainy season [81]. *M. falciger* and *M. natalensis*, both with distributions extending out of the tropics into the southern African region, will also lose a considerable part of their range. This southern African region is expected to experience greater increases in temperature and aridity than areas within the tropics, leading to more xeric biomes [78].

Many termite species can change their mound architecture and size [24,26,53,61], which can mitigate to some extent against climate conditions, and large, well-populated mounds provide a better buffer against both cold and warm temperatures [23]. When modulating temperature, chimneys can be added or sealed, mound surfaces reduced, and mound walls thickened, depending on surrounding thermal conditions [24,26]. Some species can build large mounds (e.g. *M. bellicosus* mounds regularly reach heights of 4–5 m [61], with some attaining heights of 8 m (J. Korb 2022, *pers. obs.*); *M. falciger*: mound height greater than 6 m, diameters *ca* 13.5 m [19], *M. natalensis* mounds can reach heights of 5 m [82]). The *Macrotermes* genus evolved nearly 50 Mya in a warmer environment, suggesting that they may be more adaptable to change than static predictions suggest. A closer mapping of maximum mound size and architecture for each species with environmental variables may offer insight into how mounds and their architecture may facilitate the ability of species to endure climatic challenges. There are likely to be limits, however, as these changes in climate over evolutionary time took place at a far slower rate than current changes. Furthermore, construction of new mounds may be constrained because small incipient structures are less able to buffer against extremes, necessitating more frequent re-colonization of existing, non-active large mounds.

Other social species also show responses to varying temperatures. Ants may build their underground nests deeper in response to increased temperature [83], change choice of microsite [84] or change the structure and materials used [85]. Because temperatures also vary within the nest owing to solar radiation or changes with time of day, various parts of the nest become more or less favourable, and these species (and nest interlopers) tend to respond by choosing certain parts of the nest, if available (as in the case of sociable weaver nests; [78,86]) or move broods around to track optimal temperatures [84].

For *Macrotermes,* the relationship with *Termitomyces* may also allow some adaptation. Given that some *Macrotermes* species may associate with different *Termitomyces* symbionts (e.g. [11,28]), there could be selection for specific associations as climate changes. For instance, association with fungal strains able to cope with variable conditions or the new environmental conditions might be favoured. In general, we expect that termite species that are flexible in their fungal partnership might be more likely to survive fast-changing ambient conditions.

Surrounding vegetation can also influence internal conditions [60], ultimately affecting colony survival and reproduction [61]. Whether species are already at their limits, or whether there will be selection for a combination of mounds of certain architectures, with *Termitomyces* fungus able to tolerate higher temperatures, and in habitats with more shade as climate change unfolds, is unknown.

## 8. Loss of termite-facilitated ‘buffers’ at the landscape scale

Through their creation of landscape heterogeneity, termites can buffer against climate change impacts, and most notably, desertification [49]. Overall, our models support future contraction of ‘very suitable’ climate across millions of square kilometres for some species, at a time when temperatures will be warmer than today. In more xeric areas, the disappearance of termite species could facilitate desertification, given the role that mounds may currently play in providing microclimates at the landscape scale [58]. Bonachela *et al*. [49] provided a mechanistic model of how termite mounds could mitigate against desertification, based on two assumptions supported by empirical research: mounds (i) aid the efficiency with which plants use nutrients and (ii) facilitate water infiltration rates. Thus, termites may increase landscape robustness by increasing resistance to reduced precipitation, by slowing vegetation decline and desertification, and reducing the amount of precipitation needed for landscapes to recover from desertification. In essence, termite mounds may act as refuges for vegetation after die-off in the surrounding landscape. However, for termite species for which precipitation reduction over decadal timescales (30–50 years) leads to colony die-off and the ultimate loss of mounds from the system, any potential buffering effects may be negated [49].

In addition to buffering effects, termite contributions to nutrient cycling could change with changing climate. Recently, it was found that termite discovery of wood increased with increasing temperature [87]. However, this can only occur if termites themselves are able to cope with future climates. In our study, only a single species emerged with the potential to expand its range, but even this species (*M. vitrialatus*) faced a potentially insurmountable limiting factor: future biome-mismatch through habitat transformation. Although other non-*Macrotermes* termite species also contribute to nutrient cycling, the ecosystem services provided by the mounds of *Macrotermes* cannot be replaced [88], and there is also no guarantee that non-*Macrotermes* species would escape changes associated with climate change.

## 9. Limitations of model approaches and future research directions

Here, we used variables we considered most appropriate to capture climate niches for *Macrotermes.* However, for some species, data were lacking or insufficient, particularly for those restricted to forests, like *Macrotermes nobilis* and *Macrotermes ivorensis.* For species for which we had sufficient presence-only data, models produced outputs consistent with current knowledge. Nevertheless, simulations are broadscale and cannot capture finer-scale detail, e.g. habitat structure produced by surrounding vegetation (removal of which could make maintenance of internal mound temperatures more difficult, with effects on survival and reproduction; [60,61]), or finer-scale patterns in precipitation or inundation (which determine establishment success of colonies; e.g. [32,37]). In addition, other biotic factors, like the occurrence of both vertebrate and invertebrate predators, and anthropogenic disturbance can affect the occurrence of termite species [88]. Future research should consider these modulating effects to understand termite species' distributions at the landscape scale.

Temporal aspects can also limit the accuracy of model output. Much of the termite presence data we use were assembled by Ruelle [66], mainly based on museum specimens, some of which had been collected as far back as the turn of past century. The lack of more recent data emphasizes a need for updated species occurrence data. Although we used data stretching back many decades, the rate of climatic change is accelerating [89]. Conceivably, changes in the first half of the twentieth century may have had less impact than future changes in climate will have. Although we identified various data sources, none can be considered comprehensive, given that data on *Macrotermes* (and invertebrates in general) can be further complicated by issues of taxonomy and sampling [63].

Regarding SDMs, we note that despite models demonstrating good fit, accuracy depends on extant knowledge of a given species, and output is only as robust as the simulation's input data [90]. Lastly, our models do not consider species dispersal ability, which is poor for termites, or anthropogenic habitat transformation, predicted to be marked in Africa (the continent expected to experience the greatest increase in human population in coming decades [91]). In Africa, forests, woodland and savannahs are being converted to cropland and rangeland. Typically, the woody component of savannahs is harvested for building and fuel, and often fires are set to increase the grassy component, creating rangeland for livestock, which can have long-lasting effects [92]. Both forms of habitat transformation change ecosystem structure and function. Thus, our projections for future range are likely too optimistic, as they do not include the losses to anthropogenic habitat transformation. Future research could usefully quantify how current and future land use change may further restrict the distributions and population densities of species.

## 10. Conclusion

Our modelling results predict large-scale shifts in the distribution of *Macrotermes* species. Three woodland savannah species will likely lose many millions of square kilometres of ‘very suitable’ climate. The forest-restricted species, *M. muelleri*, which seems less affected by climate change, will likely lose much of its habitat through anthropogenic transformation, and the single species predicted to expand its range (*M. vitrialatus*) will be faced with a mismatch of biomes. As biome is the most important driver for this species, the potential for expansion may be curtailed. Overall, it is difficult to predict what the loss of the most suitable area will mean for *Macrotermes* or the landscapes in which they occur. Their likelihood of persisting will depend on their ability to adapt and survive in suboptimal habitats. Possible strategies include termites changing their ranges, their mound architecture, and cultivating different fungal strains. Loss of *Macrotermes* will impact the roles not provided by other termites or biota, particularly those that arise because of the large mounds themselves [88]. Among the most important of these is the creation of habitat heterogeneity on which many other species depend [3,4,44,48], with impacts on species diversity. This is the case not only for *Macrotermes,* but also for other species whose nests provide resources and thermal refuges for other species, for example, those of sociable weavers (*P. socius* [3,5,6]). Moreover, range-contraction will likely exacerbate the drying effects of climate change, with loss of microclimates and loss of buffering against desertification. It is not unreasonable under this scenario to anticipate disruption to ecosystem pattern and process, and the unfolding of ecological cascades at the landscape, and even continental scale.

## Data Availability

All data are provided in the electronic supplementary material [93].
